# Evaluation of fingerstick blood point-of-care testing of hepatitis B DNA for enhanced hepatitis B treatment decision making: a diagnostic accuracy study

**DOI:** 10.1128/jcm.01405-25

**Published:** 2026-01-20

**Authors:** Behzad Hajarizadeh, Jacob George, Miriam T. Levy, Ian Wong, Jess Howell, Gesalit Cabrera, Elise Tu, Marianne Martinello, Tanya L. Applegate, Gail V. Matthews

**Affiliations:** 1 The Kirby Institute, University of New South Wales (UNSW) 7800, Sydney, Australia; 2 Storr Liver Centre, The Westmead Institute for Medical Research, Westmead Hospital and University of Sydney, Sydney, Australia; 3 Liverpool Hospital; South Western Clinical School, University of New South Wales (UNSW) 7800, Sydney, Australia; 4 Blacktown Hospital, Sydney, Australia; 5 St Vincent’s Hospital Melbourne, Melbourne, Australia; 6 Department of Medicine, University of Melbourne, Melbourne, Australia; 7 Department of Epidemiology and Preventive Medicine, Monash University 2541, Melbourne, Australia; 8 The Burnet Institute, Melbourne, Australia; 9 Prince of Wales Hospital, Sydney, Australia; 10 St Vincent’s Hospital Sydney, Sydney, Australia; Mayo Clinic Minnesota, Rochester, Minnesota, USA

**Keywords:** HBV, HBV DNA, viral load, diagnostics, linkage to care, sensitivity, specificity

## Abstract

Hepatitis B virus (HBV) DNA testing is essential for the management of HBV infection. Routine HBV DNA tests in central laboratories are expensive and require processed venous blood, limiting accessibility. This study is the first published assessment of the point-of-care Xpert HBV DNA assay performance using fingerstick capillary blood compared with standard-of-care venous blood testing. Participants with chronic HBV infection were enrolled from six hospitals. Fingerstick capillary blood was tested using Xpert HBV Viral Load assay (quantification lower limit: 100 IU/mL). Venipuncture whole blood was tested with COBAS AmpliPrep/COBAS TaqMan HBV DNA Test (gold standard). The sensitivity and specificity of the Xpert were evaluated for identifying HBV DNA ≥100 and >2,000 IU/mL. Agreement between quantitative measurements of assays was assessed. A total of 246 participants were included (median age 45, 46% female, 18% HBeAg positive, 48% on HBV treatment, 6% with cirrhosis). For HBV DNA ≥100 IU/mL, the sensitivity and specificity of the Xpert were 97.0% (95% CI: 94.9, 99.1) and 90.3% (86.6, 94.0), respectively. For HBV DNA >2,000 IU/mL, the sensitivity and specificity were 95.3% (92.7, 98.0) and 95.0% (92.4, 97.8), respectively. Viral load differences in non-concordant samples ranged from 0.1 to 1.1 log IU/mL. Overall, the Xpert viral loads were a mean 0.12 log IU/mL higher than gold standard (95%CI: −0.43, 0.67). In conclusion, minimal differences in HBV DNA levels were identified between the Xpert and gold standard assays, with differences in non-concordant results unlikely to impact clinical decisions. This evidence supports developing a dedicated Xpert HBV DNA fingerstick assay for decentralized care, crucial for remote, resource-limited settings and hard-to-reach populations, including prenatal care for women with HBV.

**IMPORTANCE:**

This study represents the first assessment of a point-of-care hepatitis B virus (HBV) DNA assay using fingerstick capillary blood (Xpert HBV Viral Load assay). Our findings demonstrated high sensitivity and specificity for the point-of-care test, with close agreement between the point-of-care and standard-of-care assays across the full quantitative spectrum of HBV viral load measurements. Importantly, the differences between the assays in participants with non-concordant results were not substantial enough to alter clinical management, suggesting that this point-of-care method is both accurate and reliable for clinical use. By highlighting the potential for decentralizing HBV care, our research provides compelling evidence to support the development of a dedicated Xpert HBV DNA point-of-care test. Such a development could greatly benefit patients in remote and resource-limited settings, where access to laboratory-based testing is limited.

## INTRODUCTION

Hepatitis B virus (HBV) infection is a global public health concern despite the availability of effective, well-tolerated, and affordable therapy (1). The World Health Organization (WHO) aims to eliminate HBV *as a public health threat* by 2030, with specific targets set of 90% of people living with chronic HBV diagnosed and 80% of those eligible for antiviral therapy on treatment (2). However, of an estimated 258 million people living with chronic HBV infection in 2022 globally, only 14% have been diagnosed and 8% of those eligible were receiving treatment (3). A modeling study projected that no country is currently on track to meet the 2030 HBV elimination targets (4).

Although the diagnosis of HBV infection is primarily based on serologic testing for HBV surface antigen (HBsAg), HBV DNA quantification tests—the current gold standard for assessing viral replication—are essential in clinical management of chronic HBV infection. HBV DNA assays are used for determining eligibility for antiviral treatment and evaluating treatment response, and are recommended by all major HBV clinical guideline as part of the clinical decision-making algorithm (5–8). However, testing remains sub-optimal in most countries (9–14), with WHO recognizing lack of HBV DNA testing as a major gap in the HBV clinical care pathway (5). One of the main factors contributing to low uptake of HBV DNA testing is limited access, particularly in remote areas or resource-limited settings, given high cost, infrastructure requirements, and logistical support (15–18). Standard-of-care quantitative HBV DNA tests require referral for blood collection, preparation of plasma, and centralized well-equipped molecular laboratories with large, automated platforms. Another barrier to standard-of-care HBV DNA testing, even in well-resourced settings, is the multi-visit process required for patients to receive their results, which prolongs time to commence therapy if they are eligible. An easy-to-run, affordable HBV DNA test at the point-of-care using a simple fingerstick sample could overcome these barriers and expand global access to HBV testing and treatment (5, 18, 19).

The GeneXpert diagnostic system provides a generic fully integrated and automated platform for sample preparation, nucleic acid amplification, and real-time polymerase chain reaction (PCR)-based target detection. It can be conducted outside a laboratory setting and provides results within 90 min, depending on the test. GeneXpert is widely used globally for diagnosis and clinical management of infectious diseases, such as hepatitis C (HCV), HIV, tuberculosis, and acute respiratory viruses (20–22). The Xpert HBV Viral Load test, which uses plasma samples collected via venipuncture has received both European CE-IVD and Australian Therapeutic Goods Administration certification. Although this assay has demonstrated high diagnostic performance in several studies (23–28), the requirement for a trained phlebotomist and a sample centrifugation system significantly limits its operation by non-laboratory trained professionals, a major impediment for the potential integration of the test into outpatient, primary health care or community clinics and other decentralized settings or in remote areas. A simplified fingerstick-based point-of-care testing model could effectively overcome this barrier. We conducted this study as the first global validation of the diagnostic performance of the Xpert HBV Viral Load using fingerstick capillary blood samples at the point-of-care compared with a standard-of-care laboratory-based assay using venous blood plasma samples.

## MATERIALS AND METHODS

This study is reported in accordance with the STARD guidelines for reporting diagnostic accuracy studies (29).

### Study design, setting, and participants

This prospective study was conducted across six hospital-based liver disease clinics in Australia. Adults (≥18 years old) who were diagnosed with chronic HBV infection were eligible for enrollment, irrespective of their HBV DNA levels, HBV e-antigen (HBeAg) status, or anti-viral treatment. Chronic HBV was defined as a positive HBsAg test result persisting for more than 6 months. At each site, consecutive patients who met the eligibility criteria and were willing to participate were enrolled. The recruitment period spanned from 16 November 2022 to 7 June 2024. Informed consent was obtained from each participant, and they received a shopping voucher valued at AUD 30 as reimbursement. All research was conducted in accordance with both the Declarations of Helsinki and Istanbul.

### Test methods

After enrollment and prior to HBV DNA testing, demographic and clinical data were collected, including age, sex, ethnicity, country of birth, HBeAg status, alanine transaminase (ALT) levels, cirrhosis status, and current HBV clinical management.

Then, from each participant, one fingerstick capillary blood sample and one venipuncture whole blood sample were collected and tested as follows:

- **Point-of-care HBV DNA quantification:** Fresh capillary blood was collected by piercing the participant’s finger with a MiniCollect safety lancet (Greiner BioOne, Monroe, Frickenhausen, Germany). To minimize potential interference from interstitial fluid, the first drop of blood was wiped away, and the subsequent drop was used for analysis. All procedures were performed in adherence to best practice in capillary blood sampling technique. A minivette (Minivette POCT 100 μL; Sarstedt, Nümbrech, Germany) was used to transfer 100 μL of the capillary blood into a specimen vial containing 900 μL of the supplied buffer (Cepheid, Sunnyvale, CA, USA), resulting in a 1:10 dilution. This step was performed to solubilize DNA from whole blood, optimize subsequent automated processing and DNA quantification, and ensure sufficient volume for loading. Using a 1000 μL precision pipette, the contents were mixed and loaded into the sample chamber of the Xpert HBV Viral Load Assay (Cepheid, Sunnyvale, CA, USA; lower limit of quantification: 10 IU/mL = 1 log IU/mL; range of quantification: 10 to 10^9^ IU/mL = 1 to 9 log IU/mL), with the HBV DNA test performed on the GeneXpert IV instrument system in real time on-site. The original HBV DNA level reads were adjusted to address the dilution (more details provided in the Data analysis section). This test uses reverse transcriptase PCR and provides a measure of HBV viral load within 60 min. The GeneXpert IV was operated by a trained nurse following the manufacturer’s instructions at the outpatient clinic.
- **Standard-of-care HBV DNA quantification:** A venous whole blood sample was collected in the hospital on the same day as the point-of-care test and 0.6 mL tested in the laboratory using the COBAS AmpliPrep/COBAS TaqMan HBV DNA Test (Roche Diagnostics, Basel, Switzerland; lower limit of quantification: 20 IU/mL = 1.3 log IU/mL; range of quantification: 20 to 1.8 × 10^8^ IU/mL = 1.3 to 8.3 log IU/mL) according to manufacturer’s instructions. This test was conducted at the hospital laboratory or pathology unit.

In cases when no valid results were provided on the Xpert HBV Viral Load assay (e.g., technical issue, error/invalid message on the machine), the point-of-care test was repeated using a new fingerstick sample, provided the participant was still on-site and willing to provide an additional sample. Participants received clinical services based on their standard-of-care test results, following the clinical guidelines (7). The results from the Xpert HBV Viral Load assay were not shared with participants, as this was a research-use only test under validation and was not approved for clinical management.

Nurses at the study sites received standardized training on point-of-care testing procedures and dilution protocols. Uniformity in fingerstick collection was maintained across all sites by using the same consumables, including MiniCollect safety lancet and Minivettes. Refresher training was available upon request to ensure continued adherence to the protocol.

### Data analysis

The standard-of-care COBAS AmpliPrep/COBAS TaqMan HBV DNA Test was considered as the gold standard. The agreement of the Xpert HBV Viral Load Assay with the standard-of-care was assessed among participants for whom results from both assays were available. The agreement was assessed by (i) evaluating sensitivity and specificity for detection of HBV DNA using pre-specified cut-offs and (ii) evaluating the quantitative agreement between HBV DNA levels measured by the two assays. Given that fingerstick samples were diluted 1:10 for testing with the Xpert HBV Viral Load Assay and to enable direct comparison with standard-of-care, the original HBV DNA levels reported on Xpert were multiplied by 10 and the adjusted values were used in the analysis. This adjustment was conducted as an additional step in the validation process for this research-use-only test and required manual calculation outside of the Xpert system.

- **Sensitivity and specificity:** The diagnostic performance of the Xpert assay was assessed for two outcomes: (i) identifying those with quantifiable HBV DNA and (ii) identifying those with HBV DNA >2,000 IU/mL (=3.3 log IU/mL). To align with the 1:10 dilution adjustment for Xpert, the lower limit of quantification for the adjusted viral load was set at 100 IU/mL (=2 log IU/mL) for comparative analyzes. Therefore, in the first and second analysis, the sensitivity and specificity of the Xpert assay were evaluated for HBV DNA ≥100 IU/mL (vs <100 IU/mL or undetectable), and for HBV DNA ≥2,000 IU/mL (vs <2,000 IU/mL or undetectable), respectively. The second HBV DNA cut-off was chosen based on the recent WHO recommendations for treatment of all people with HBV DNA >2,000 IU/mL and elevated ALT (5). Assuming a prevalence of quantifiable HBV DNA of 35% and a sensitivity and specificity of 100%, a sample size of 246 would provide a 95% CI of 29%–41% for the prevalence estimate and 95% CIs of 95.8%–100.0% and 97.7%–100.0% for the estimates of sensitivity and specificity, respectively.
- **Quantitative agreement:** Among participants with quantifiable HBV DNA by both assays, we generated a Bland–Altman difference plot to assess bias and agreement of HBV DNA levels measured by the the Xpert assay compared to the gold standard. Pearson’s correlation was also used to evaluate the linear correlation between HBV DNA levels measured by the two assays.

## RESULTS

### Participants

**Fig 1:**
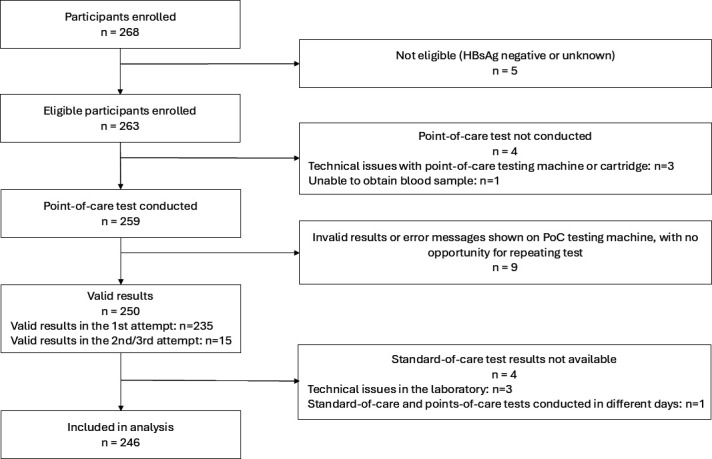
Overview of the participants’ flow.

A total of 268 participants were enrolled, of whom five did not meet eligibility criteria. Of 263 eligible participants, 17 were excluded for the following reasons: no point-of-care tests conducted (*n* = 4), no valid point-of-care test results (*n* = 9), and no standard-of-care test results (*n* = 4). More detailed reasons for participant exclusion are provided in Fig. 1. Ultimately, 246 participants with results from both assays conducted on the same day were included in the analysis (Fig. 1).

Among the 246 participants included in the analysis, the median age was 45 years (IQR: 37, 59), 46% were female, 93% were born outside Australia—the overwhelming majority (70%) in East or South-East Asia, and one participant identified as Torres Strait Islander (Indigenous Australian). Over a third of participants (38%) were receiving HBV anti-viral therapy, either tenofovir or entecavir. Most participants (63%) had elevated ALT, 18% were HBeAg positive, and 6% had cirrhosis (Table 1).

**TABLE 1:** Baseline demographic and clinical characteristics of participants

Baseline characteristics	Total (*n* = 246)
Age, median (IQR)	45 (37, 59)
Sex, *n* (%)	
Male	131 (53)
Female	114 (46)
Transgender	1 (<1)
Region of birth, *n* (%)	
Australia	18 (7)
East or South-East Asia	172 (70)
South Asia or Middle-East	24 (10)
Africa	16 (7)
Other	16 (7)
Currently pregnant (among women), *n* (%)	
No	104 (91)
Yes	10 (9)
Elevated ALT^*a*^, *n* (%)	
No	92 (37)
Yes	154 (63)
HBeAg, *n* (%)	
Negative	198 (80)
Positive	45 (18)
Unknown	3 (1)
Cirrhosis, *n* (%)	
No	218 (89)
Yes	14 (6)
Unknown	14 (6)
Current HBV clinical management, *n* (%)	
Initial assessment	34 (14)
Monitoring only	118 (48)
Treatment	93 (38)
Other^*b*^	1 (<1)
HIV co-infection, *n* (%)	
No	225 (91)
Yes	7 (3)
Unknown	14 (6)
HCV co-infection, *n* (%)	
No	230 (94)
Yes	3 (1)
Unknown	13 (5)

^*a*^ ALT threshold: 19 and 30 IU/L for women and men, respectively.

^*b*^ Self-discontinued treatment.

### Test results

Based on the quantification thresholds of the standard-of-care test (i.e., 20 IU/mL), HBV DNA was undetectable in 60 participants (24%), detectable but lower than 20 IU/mL (<1.3 log) in 31 participants (13%), between 20 and 1.8 × 10^8^ IU/mL (1.3 to 8.3 log) in 147 participants (60%), and higher than 1.8 × 10^8^ IU/mL (>8.3 log) in 8 participants (3%). Among those with HBV DNA between 20 and 1.8 × 10^8^ IU/mL, median HBV viral load was 910 IU/mL (2.9 log), with an IQR of 225 to 7,920 IU/mL (2.3 to 3.9 log; Fig. S1).

For identification of samples with quantifiable HBV DNA (≥100 IU/mL or ≥2 log IU/mL), the sensitivity of the Xpert HBV Viral Load assay was 97.0% (95% CI: 94.9, 99.1) and the specificity was 90.3% (95% CI: 86.6, 94.0; Table 2). In 15 of 246 samples (6%), the HBV viral loads detected by the two assays were non-concordant (i.e., one assay reporting ≥100 IU/mL and the other reporting <100 IU/mL, or vice versa). In these samples, the viral load difference ranged between 38 and 388 IU/mL (0.2 to 1.1 log; Table 3). In all these cases, the Xpert gave a slightly higher reading than the standard-of-care.

**TABLE 2:** Number of samples identified with quantifiable HBV DNA (≥100 IU/mL or ≥2.0 log IU/mL) versus unquantifiable HBV DNA (<100 IU/mL or <2.0 log IU/mL) by the point-of-care Xpert HBV viral load assay and the standard-of-care assay

		Standard-of-care		
		HBV DNA ≥100 IU/mL	HBV DNA <100 IU/mL^*a*^	Total
Xpert	HBV DNA ≥100 IU/mL	129	11	140
	HBV DNA <100 IU/mL^*a*^	4	102	106
	Total	133	113	246

^*a*^ Included samples with HBV DNA identified as undetectable, or detectable but lower than 100 IU/mL (<2.0 log).

**TABLE 3:** Characteristics of participants with non-concordant HBV viral loads detected by the two assays, using 100 IU/mL (2.0 log) and 2000 IU/mL (3.3 log) as the thresholds^*a*^

ID	Sex	HBeAg	ALT (IU/L)	HBV clinical management	HBV viral loads detected by standard-of-care assay (IU/mL)	HBV viral loads detected by point-of-care Xpert assay (IU/mL)	Difference in HBV viral loads detected by two assays (log IU/mL)
100 IU/mL or 2.0 log IU/mL as the threshold							
210-028	F	Neg	30	Initial assessment	63	230	0.6 log
216-007	M	Pos	44	Treatment	93	160	0.2 log
216-027	F	Neg	66	Monitoring only	48	210	0.6 log
216-042	F	Pos	20	Treatment	80	210	0.4 log
216-043	F	Neg	31	Monitoring only	55	100	0.3 log
216-045	F	Neg	14	Treatment	72	110	0.2 log
216-048	F	Neg	29	Monitoring only	92	160	0.2 log
216-056	M	Pos	29	Treatment	32	420	1.1 log
302-035	M	Neg	31	Monitoring only	86	210	0.4 log
202-034	M	Neg	28	Monitoring only	Detected, <20	260	–
216-005	M	Unknown	50	Treatment	Detected, <20	150	–
202-035	M	Neg	17	Treatment	198	Detected, <100	–
202-041	M	Neg	34	Monitoring only	115	Detected, <100	–
210-012	F	Pos	22	Initial assessment	115	Detected, <100	–
216-086	F	Neg	32	Monitoring only	108	Detected, <100	–
2,000 IU/mL or 3.3 log IU/mL as the threshold							
202-042	F	Pos	27	Monitoring only	1,970	2,610	0.1 log
210-006	F	Neg	23	Monitoring only	1,760	2,020	0.1 log
216-003	F	Neg	22	Monitoring only	976	2,110	0.3 log
216-023	F	Neg	15	Monitoring only	821	2,320	0.5 log
216-026	M	Neg	27	Monitoring only	1,730	7,660	0.6 log
216-034	F	Neg	26	Monitoring only	1,310	3,510	0.4 log
216-046	M	Neg	48	Monitoring only	755	3,840	0.7 log
216-067	M	Neg	26	Monitoring only	383	2,510	0.8 log
216-068	F	Neg	29	Monitoring only	1,640	2,550	0.2 log
302-002	M	Neg	14	Initial assessment	2,250	1,100	−0.3 log
302-008	F	Neg	16	Monitoring only	2,610	1,770	−0.2 log
302-036	M	Neg	29	Monitoring only	2,800	1,720	−0.2 log

^*a*^ –, not applicable.

For identification of samples with HBV DNA >2,000 IU/mL (>3.3 log IU/mL), the sensitivity of the Xpert HBV Viral Load assay was 95.3% (95% CI: 92.7, 98.0), and the specificity was 95.0% (95% CI: 92.4, 97.8; Table 4). In 12 samples, the viral loads detected by the two assays fell in different categories using this threshold (i.e., one assay reporting >2,000 IU/mL and the other reporting ≤2,000 IU/mL, or vice versa). In these samples, the absolute viral load difference ranged between 260 and 5930 IU/mL (0.1 to 0.8 log; Table 3). In 9 of 12 cases, the Xpert-reported values were slightly higher than the standard-of-care.

**TABLE 4:** Number of samples identified with HBV DNA >2,000 IU/mL (>3.3 log) versus ≤2,000 IU/mL (≤3.3 log) by the point-of-care Xpert HBV viral load assay and the standard-of-care assay

		Standard-of-care		
		HBV DNA >2,000 IU/mL	HBV DNA ≤2,000 IU/mL^*a*^	Total
Xpert	HBV DNA >2,000 IU/mL	61	9	70
	HBV DNA ≤2,000 IU/mL^*a*^	3	173	176
	Total	64	182	246

^*a*^ Included samples with HBV DNA identified as undetectable or detectable but at the level of 2,000 IU/mL (3.3 log) or lower.

**Fig 2:**
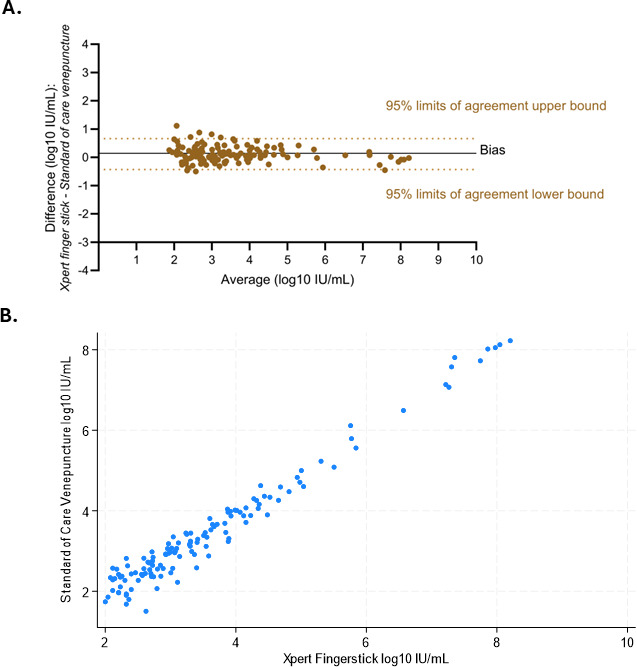
(**A**) Bland–Altman bias plot of differences in HBV viral loads detected by the point-of-care Xpert HBV viral load assay and the standard-of-care assay (*n* = 130, bias: 0.118 log IU/mL, 95% limits of agreement: 0.430 to 0.666 log IU/mL); (**B**) linear correlation between HBV viral loads detected by the point-of-care Xpert HBV viral load assay and the standard-of-care assay (*n* = 130, *r* = 0.9824, *P* < 0.001).

Among the samples with quantifiable HBV DNA levels detected by both assays (*n* = 130), the Bland–Altman plot identified that the HBV viral loads detected by the Xpert assay were a mean of 0.12 log IU/mL higher than those detected by the standard-of-care assay, with the 95% limits of agreement between –0.43 and 0.67 log IU/mL (Fig. 2A). Back-transforming these values, the mean difference corresponds to a ratio of 1.32 (95% limits of agreement: 0.37 to 4.70), indicating that the Xpert assay reported HBV DNA levels, on average, 32% higher than the standard-of-care assay. The Pearson’s correlation test showed a strong linear correlation between HBV DNA levels measured by the two assays (*r* = 0.9824, *P* <0.001; Fig. 2B).

In 11 samples, the difference between results by two assays was higher than 0.5 log IU/mL, including only one sample with results differing by more than 1.0 log IU/mL (1.1 log).

## DISCUSSION

In this first validation, we found excellent correlation between HBV viral loads, measured by the Xpert HBV DNA fingerstick assay (as a sample type), and the gold standard assay using venous blood. High sensitivity and specificity were demonstrated at both chosen viral load cut-offs (100 IU/mL and 2,000 IU/mL), and close agreement was observed between HBV viral load measurements obtained from the two assays across the quantitative spectrum. These findings provide evidence to support the development of a dedicated Xpert HBV DNA point-of-care assay using fingerstick capillary blood to simplify and decentralize HBV clinical care, particularly in remote and resource-limited settings.

Our study demonstrated extremely high concordance (*r* = 0.9824) for the Xpert HBV DNA fingerstick assay versus venous blood. In all non-concordant cases, the standard-of-care test gave a result slightly lower than the Xpert test, hence the reduction in specificity. In clinical practice, an HBV DNA quantification threshold is used to evaluate viral suppression or to recommend anti-viral treatment under specific circumstances (e.g., cirrhosis or significant liver fibrosis, HIV or HCV co-infection, extrahepatic manifestations) (5, 6, 8). For determining treatment eligibility, the sensitivity of an HBV DNA test is typically prioritized to ensure that no patients who would benefit from treatment are missed. Suboptimal specificity may lead to overtreatment in some patients who would not otherwise be eligible but is unlikely to compromise patients’ health, given the minimal side effects of HBV antiviral therapies. In our study, only two patients who had a detectable HBV viral load >100 IU/mL by the Xpert had a viral load <20 IU/mL by the standard-of-care assay and would therefore have been ineligible for treatment by current testing algorithms. Our findings, therefore, demonstrating high sensitivity (97%) with somewhat lower specificity (90%) of the Xpert, support the use of this assay in clinical practice. To the best of our knowledge, only one other study is being conducted with similar objectives. In that study, conducted among 100 people with chronic HBV in Senegal, a preliminary analysis reported a similarly high performance of the Xpert HBV DNA fingerstick assay (30).

The sensitivity and specificity of the Xpert HBV DNA fingerstick assay were also high (95%) at the HBV DNA threshold of 2,000 IU/mL (3.3 log), recommended by WHO and other international guidelines for commencing treatment (5, 8). The viral load difference between samples was less than 1.0 log for all participants with non-concordant results. This difference does not usually change clinical decision-making in real-world practice, given expected fluctuations in HBV DNA levels over time (31–33). These findings further support the use of this assay for assessing treatment eligibility in clinical settings.

Across all participants with quantifiable HBV DNA, our findings demonstrated a high agreement between HBV viral loads measured by the two assays. The viral load measurements by the Xpert assay were a mean of 0.12 log IU/mL higher than those detected by the standard-of-care assay, with a difference higher than 1.0 log IU/mL observed in only one sample. In this study, we minimized the potential for pre-analytical variability related to sample quality by adhering to best practice in capillary blood sample collection technique, thereby reducing the impact of sample quality on clinical interpretation of results. However, variations in viral loads detected by different assays have been reported in several studies, even when using the same sample types. For example, studies comparing different assays using plasma or serum from venous blood, reported mean differences ranging from 0.11 to 0.31 log between assays (34–36). In a study comparing Abbott RealTime and TaqMan assays, a difference of 1.0 log IU/mL between two assays was reported in 9% of the samples (34).

While HBV diagnosis relies primarily on HBsAg serologic testing, HBV DNA quantification remains critical for assessing viral replication and guiding treatment decisions in chronic infection (5–8), typically requiring referral to a centralized well-equipped molecular laboratory in most settings. A large majority of people with chronic HBV are living in low/middle-income countries (3), where access to a centralized laboratory or specialist HBV clinical facilities is often limited (18), a key factor contributing to low uptake of HBV DNA testing and treatment (15, 17). Limited access is also a major barrier for people in remote areas in high-income countries. In Australia, for example, the HBV DNA testing uptake was lower among people with chronic HBV living in remote areas (9), including among pregnant women (37). Given limited access to standard-of-care HBV DNA testing globally, WHO recommended strategies, such as point-of-care and reflex testing among those with a positive HBsAg test, to improve HBV DNA testing uptake to facilitate patient management (5). Nevertheless, in seeking to simplify treatment guidelines, WHO has also recommended that when HBV DNA testing is unavailable, treatment may be considered based on persistently abnormal ALT (5). In pregnancy, treatment for all women with chronic HBV in the third trimester has also been advised if HBV DNA testing is unavailable (5). However, given multiple other causes for liver function abnormalities and uncertainty about the acceptability of this approach in pregnancy, it remains unclear how successfully this recommendation will be implemented.

Point-of-care diagnostic technologies have been successfully integrated into various health care settings, including in remote areas, community and primary health care clinics, and outreach programs for people not engaged in mainstream healthcare pathways (38–40). It is therefore reasonable to suggest that HBV DNA point-of-care testing has the same potential to broaden access, increase testing uptake, and enhance treatment initiation, given the crucial role of HBV viral load in decision-making for treatment initiation. A recent systematic review reported data from six studies (all in low/middle-income countries), which utilized point-of-care HBV DNA assays using venous blood or dried blood spot samples in HBV care facilities (41). In these studies, a pooled estimate of 84% of people with chronic HBV were tested for HBV DNA, with 88% of those eligible for anti-viral treatment commencing therapy (41). A point-of-care assay using a small volume of fingerstick blood has the potential to further facilitate decentralized testing and linkage to care at the point of care, by eliminating the needs for phlebotomy and sample centrifugation. Future implementation trials are needed to evaluate their usage, including both feasibility and acceptability.

Some potential barriers to the large-scale implementation of HBV DNA point-of-care testing models include sustainable funding for the costs of the device and test kits, simplified regulatory and fit-for-purpose accreditation pathways, trained point-of-care testing workforce, and ongoing technical and quality assurance support from specialist point-of-care testing network providers (38, 42, 43). For the Xpert, this capacity has already been established in many countries (mostly low/middle-income countries), given the widespread use of the GeneXpert platforms for HIV and tuberculosis testing (17), and more recently for HCV testing (44, 45). The development of HBV DNA assays for this platform provides an opportunity for health services to leverage an existing framework to increase access to testing, linkage to care and support for people with chronic HBV. Further, the recent emphasis from WHO on triple elimination supports the integration of HBV point-of-care testing into antenatal programs and health services (46)

Clinical guidelines often require elevated ALT as another criterion for initiating HBV treatment (5–8). A point-of-care test for ALT would complement point-of-care HBV DNA testing, enabling more comprehensive and guideline-aligned decision-making. Although no point-of-care ALT assay is currently available for clinical use, studies have shown promising preliminary findings, with further studies ongoing (47, 48). Such a model, which could include both point-of-care ALT and HBV DNA testing to guide decision making in a variety of settings, could be truly transformative in the global HBV treatment landscape, greatly enhancing progress towards viral hepatitis elimination goals.

This study had several limitations. Data on HBV genotypes were unavailable for participants; therefore, we were unable to compare the performance of the Xpert assay across HBV genotypes. Given the ethnicity distribution in our study population, we would anticipate genotype B and C in most participants. We also did not conduct a stratified analysis by HBeAg status, given the small number of HBeAg positive samples. The number of samples with very high HBV viral load (>6 log IU/mL) was also small, which limited our analysis on agreement between the two assays at such high HBV DNA levels. However, this is not a major concern in clinical practice since anticipated differences in quantitation at high HBV DNA levels would not impact treatment eligibility criteria. We did not compare the Xpert fingerstick to Xpert venous blood assays in this study. Such a study could evaluate the extent of discordance due to using fingerstick blood, which could be overcome by calibration. The reproducibility of the Xpert assay was not evaluated in this study. Future studies are needed to assess the assay reproducibility and the potential impact of variability associated with capillary blood collection.

In conclusion, this study demonstrated excellent performance of the point-of-care Xpert assay for the quantitation of HBV DNA from fingerstick capillary samples. These findings support the development of a dedicated Xpert HBV DNA fingerstick assay, which holds significant potential to facilitate decentralized HBV testing and treatment services. This model could greatly benefit people living with HBV in low/middle-income countries with limited resources, as well as those living in remote areas. Further studies are needed to evaluate the impact of using the point-of-care Xpert assay with fingerstick blood on the uptake of HBV DNA testing and anti-viral treatment.

## SUPPLEMENTAL MATERIAL

Figure S1 jcm.01405-25-s0001.docx Distribution of HBV viral loads detected by the standard-of-care assay.

ASM does not own the copyrights to Supplemental Material that may be linked to, or accessed through, an article. The authors have granted ASM a non-exclusive, world-wide license to publish the Supplemental Material files. Please contact the corresponding author directly for reuse.
