# Supplementary material for: Evaluation of fingerstick blood point-of-care testing of hepatitis B DNA for enhanced hepatitis B treatment decision making: a diagnostic accuracy study
Source: J Clin Microbiol. 2026 Jan 20;64(2):e01405-25. doi: 10.1128/jcm.01405-25 (PMC12892972; doi:10.1128/jcm.01405-25)
Supplement: Figure S1 — Distribution of HBV viral loads detected by the standard-of-care assay. [file jcm.01405-25-s0001.docx]

**Supplementary Materials**

**Supplementary Figure 1:** Distribution of HBV viral loads detected by the standard-of-care assay.

*The horizontal lines represent median and interquartile range.*
